# Genetic market in cattle (Bull, AI, FTAI, MOET and IVP): financial payback based on reproductive efficiency in beef and dairy herds in Brazil

**DOI:** 10.21451/1984-3143-AR2018-0091

**Published:** 2018-08-17

**Authors:** Pietro Sampaio Baruselli, Alexandre Henryli de Souza, Manoel Francisco de Sá, Marcio Oliveira Marques, Jose Né́lio de Sousa Sales

**Affiliations:** 1 Department of Animal Reproduction, FMVZ-USP, São Paulo, SP, Brazil.; 2 Ceva Animal Health, Paulínia-SP, Brazil.; 3 Alta Genetics, Uberaba, MG, Brazil.; 4 Geraembryo, Londrina, PR, Brasil; 5 Department of Veterinary Medicine, Universidade Federal de Lavras, Lavras, MG, Brazil.

**Keywords:** biotechnology, cows, economic, reproduction

## Abstract

A number of reproductive biotechnologies are currently available to multiply offspring from high genetic merit animals to enhance reproductive efficiency and profitability both in dairy and beef herds. Some of these technologies such as fixed time artificial insemination (FTAI), when correctly implemented, generally allow greater reproductive performance than natural breeding. Besides the use of frozen-thawed semen during artificial insemination, cattle recipients can also be synchronized to receive embryos (produced *in vivo* or *in vitro*) at set dates with fertility results that usually outperforms natural breeding as well as artificial insemination (AI), particularly during warm seasons and in repeat breeders cows. Altogether, the use of hormonal programs to synchronize ovulation time simplify field routine, can easily fix physiological limitations related to postpartum anestrus (beef cows), poor estrus detection efficiency due to less evident estrus signs (dairy cows), making AI and ET viable to commercial herds both in terms of results and economical returns.

## Introduction

Modern beef and dairy production systems are dependent upon strategies to hasten and maximize the use of reproductive biotechnologies in order to match the increasing food demand worldwide. Besides proven efficacy, these technologies must comply with easy and direct field application to improve productivity, and yet yield positive economic returns to cattle producers.

In beef cattle, profitability is generally measured by the number of calves produced within a year (shortened calving intervals, earlier births during calving season, increased calf uniformity, more concentrated calving season and heavier calves at weaning time), which are destined to meat production or herd replacement ( Baruselli *et al*., 2017a ). In dairy production systems, reproductive performance greatly affects profitability because of its direct impact mainly on the average milk production per cow per day, number of replacements produced, and rates of voluntary and involuntary culling ( Britt, 1985 ). For example, the University of Wisconsin launched back in 2010 an extension effort to improve reproductive efficiency in herds in the Midwest-USA (The ReproMoney program) and released some tools (available at http://www.dairymgt.info/tools.php ) to help producers to evaluate the impact of poor reproductive efficiency in their herds. This prediction model estimates that each point in pregnancy rate (% cows pregnant out of cows available to AI within 21d periods) for a dairy herd costs roughly $15 to $30 US per cow per year. Estimating a ballpark number for the economic impact of improving reproduction in beef and dairy operations is crucial to producers, allowing them to calculate the level of investment required to achieve better reproductive performance and compare it to expected returns. Hence, it appears obvious for modern herds that the more aggressive use of reproductive technologies are essential to maximize herd efficiency and a financially sound cattle-enterprise.

**Figure 1:**
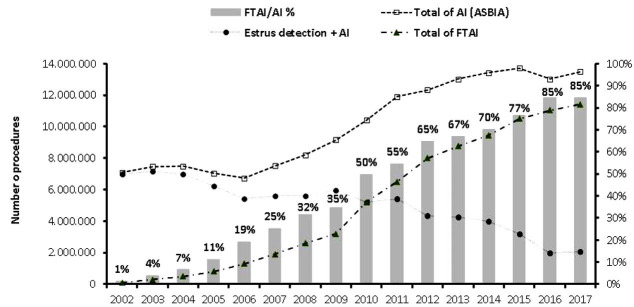
Use of artificial insemination (AI) and fixed-time AI (FTAI) from 2002 to 2017 in Brazilian cattle herds. The total number of AI procedures considered AI after estrus detection, while the numbers of FTAI are estimated based on the number of protocols sold at yearly basis (information provided by Animal Health companies) and the total AI records are based on the semen straw sales in the country (ASBIA, 2018; http://www.asbia.org.br ). Records were gathered and prepared by P.S. Baruselli, Department of Animal Reproduction, FMVZ-USP, São Paulo, SP, Brazil, 2017.

Not surprisingly, herds in several countries including Brazil have adopted fixed time artificial insemination (FTAI) to leverage their profits. Breeding records compiled in 2016 clearly shows that Brazilian beef and dairy herds still heavily utilize natural service as their main breeding system (approximately 88 to 90% of the cows are bred by bulls; Baruselli, 2016 ). Interestingly, the use of artificial insemination and embryo transfer have shown significant growth in the last 15 years. According to the Brazilian Association of Artificial Insemination (ASBIA; http://www.asbia.org.br ), the number of commercialized doses of semen increased from 7 million in 2003 to roughly 14 million in 2017. As a result, the percentage of cows and heifers that undergo artificial insemination has risen from 6% to achieve the 12% milestone in later years. This growth was mainly possible due to the widespread use of FTAI, which grew from only 1% of all AI done in the country in 2002 to astonishing 85% of AI in 2017 ( Fig. 1 ). Thus, to put it in hard count numbers, a total of 11.5 million FTAI procedures are currently performed in Brazilian herds each year – trends across the years for the use of FTAI in Brazilian herds are shown in Fig. 1 .

As expected, the increase in FTAI use and the expansion of embryo utilization by commercial herds are backed by solid literature. For example, it has been found that when FTAI is used early in the breeding season, it can clearly increase reproductive performance compared to natural service ( Baruselli *et al*., 2018 ). Similarly, the fixed-time embryo transfer (FTET) improves the proportion of recipients selected for embryo transfer in beef and dairy herds, and also produces greater pregnancy rates following transfer comparing to natural estrus detection ( Baruselli *et al*., 2010 ; Rodrigues *et al*., 2010 ). Nowadays, embryo transfer can be used to disseminate superior genetics, but most importantly, herds in warm regions utilize FTET to lessen poor conception results following natural service and/or AI generally found during heat stress seasons and in repeat breeder cows ( Baruselli et al., 2011 ).

Simple synchronization protocols, a good network of embryo labs & trained veterinarians, and the possibility to increase conception results even under severe heat stress has created a good environment for ET use in Brazil. As a result, the Brazilian embryo industry also showed a significant growth in the past 15 years, most of it associated to the adoption of *in vitro* technologies. Accordingly, in a little over a decade, the embryo market in Brazil has experienced over 5-fold increase in embryo production. Besides to this increase in numbers of produced embryos, the *in vitro* fertilization/culture has almost fully replaced the traditional *in vivo* superovulation & uterine flushing (MOET) as the technique of choice for embryo production in Brazil ( Viana *et al*., 2017 ). Thus, the *in vitro* embryo production underwent a steep increase in recent years, while numbers of *in viv*o embryo production plunged to a small share of embryo donors. However, these trends are not necessarily followed by other countries, since the IETS records showed that in 2016 production of *in vitro* embryos (IVP) were approximately 632,000 as compared to 665,000 *in vivo* derived (IVD) bovine embryos been produced world-wide ( Perry, 2017 ). In this review we present a brief report of the current state of the genetic market in Brazil, as well as discuss some aspects of the economic impact that the use of reproductive technologies can have in commercial beef and dairy herds.

## Use of FTAI in beef herds

In Brazil, the FTAI market represents approximately R$567 million (~U$175 million), with an estimate of 3,500 veterinarians directly involved with this activity. Timed AI is currently performed in approximately 8.2 million beef cows, therefore generating an increase of 8% on calf production, which represents approximately 656 thousand more calves per year or an additional income of R$820 million/year (~U$253 million) compared with natural service breeding. Timed AI also hastens parturition and adds genetic gain to commercial herds, generating an average gain of 20 kg on the weaning weight of calves, which represents 3.3 million weaned calves with extra 20 kg or, extra R$400 million (~U$123 million). Also, from weaning to slaughtering, TAI calves gain an additional 15 kg of carcass, generating extra R$482.2 million (~U$149 million). Thus, FTAI aggregates to the bovine beef chain around R$1.7 billion (more than half billion US $) per year ( Baruselli, 2016 ).

Studies were carried out by our group to evaluate the productive efficiency and the economic return of calf production systems frequently used in Brazil; the main objective was to compare natural service (88 to 90% of reproductive systems) with the FTAI followed by standard natural service with clean-up bulls ( Baruselli *et al*., 2017b ). In this study, the production system efficiency was compared under similar conditions of management and market constraints. Some of the aspects used in this simulation included Nelore bulls used for natural service in the proportion of 1 bull for 25 Nelore cows; and FTAI was set to be performed at the beginning of the breeding season using Angus semen followed by natural service with Nelore bulls (1 bull for 25 cows). Our results indicated that breeding system that uses only natural service produced only 44% of calves at the beginning of the breeding season (the first 45 days of a 90-day breeding season), compared to 75% of the system with FTAI followed by bulls (56% for FTAI and 19% for the first natural service). Also, the cows submitted only to natural service produced 8% less calves at the end of the breeding season than the cows that received FTAI followed by natural service. Moreover, FTAI anticipated calving in about 22 d compared to natural breeding FTAI. The data on calf weight at weaning and marketing value are presented in Table 1 and Table 2 . There was a significant increase in the sale price of calves produced by 100 cows from R$ 84,929.40 to R$ 106,005.40 (additional income of 25%), already discounted the operational costs with the FTAI system.

**Table 1:** Calf production (kg) and commercialization (adapted to 100 cows) generated by natural service (1 Nelore bull for 25 Nelore cows) in a 90-day breeding season.

Natural Service (100 cows)	Male	Female
Number of calves at the beginning of BS	22	22
Weaning weight (kg)	239	220
Value kg (R$)	4.93	4.60
Calf value (R$)	1,178.27	1,012.00
Sell calves (R$)	25,921.94	22,264.00
		
Number of calves at the end of BS	18	17
Weaning weight (kg)	229	210
Value kg (R$)	4.93	4.60
Calf value (R$)	1,128.97	966.00
Sell calves (R$)	20,321.46	16,422.00
		
Total number of calves (beginning + final of BS)	40	39
Sell calves (R$)	46,243.40	38,686.00
Total sales (100 cows)	R$ 84,929.40	

From Agropecuária Estrela do Céu, Lavínia, SP. 2014.

**Table 2:** Calf production (kg) and commercialization (adapted to 100 cows) generated by FTAI (Angus semen) followed by natural service (NS) with Nellore bulls (1 bull for 25 cows) in a 90-day breeding season.

TAI followed by Natural Service (100 COWS)	Male	Female
Number of calves at the beginning of BS (FTAI)	28	28
Weaning weight (kg)	269	246
Value kg (R$)	5.54	5.20
Calf value (R$)	1,490.26	1,279.20
Sell calves (R$)	41,727.28	35,817.60
		
Number of calves at the beginning of BS (NS)	10	9
Weaning weight (kg)	239	220
Value kg (R$)	4.93	4.60
Calf value (R$)	1,178.27	1,012.00
Sell calves (R$)	11,782.70	9,108.00
		
Number of calves at the end of BS (NS)	6	6
Weaning weight (kg)	229	210
Value kg (R$)	4.93	4.60
Calf value (R$)	1,128.97	966.00
Sell calves (R$)	6,773.82	5,796.00
		
Total number of calves (beginning + final of BS)	44	43
Sell calves (R$)	60,283.80	50,721.60
Total sales (100 cows)	R$ 111,005.40	
Cost of TAI for 100 cows (protocol, semen and services, R$ 50,00 per cow)	R$ 5,000.00	
Net revenue (sale of calves - cost of FTAI)	R$ 106,005.40	

From Agropecuária Estrela do Céu, Lavínia, SP. 2014.

The impact of FTAI is also cumulative throughout the years. It was observed that in suckled beef *Bos taurus* and *Bos taurus* crossbred cows exposed to FTAI weaned a calf during the subsequent breeding season (84%) compared to cows exposed to natural service (78%). In addition, survival analysis demonstrated that the mean days to calving from initiation of the calving season is shorter for TAI (26.8 ± 0.8 days) than natural service (31.3 ± 0.8 days). In addition, weaning weights for calves originated from FTAI (213.1 ± 3.7 kg) was greater than calves from natural service (200.8 ± 3.6 kg). Therefore, the use of FTAI resulted in US$ 49.14 advantage over natural breeding, as reported elsewhere (Rodgers, *et al*., 2012). Another study was carried out to evaluate the economic payback of FTAI followed by resynchronization with a second FTAI (resynchronization) compared to the FTAI followed by the natural service ( Baruselli *et al*., 2017b ). The results showed that the cost of pregnancy including the resynchronization strategy (adding a second FTAI) is lower when compared to FTAI followed by the natural service for clean-up breeding during the breeding season (R$ 121.59 *vs*. R$ 167.97). Thus, the use of resynchronization is also financially positive in beef herds.

Based on the previous assumption that resynch could be an interesting option for beef herds and to model a more aggressive FTAI approach, a further study from our research group aimed to evaluate the reproductive efficiency and the pregnancy cost for Nelore cows submitted to three consecutive FTAI programs. A total of 1,505 multiparous cows received the same FTAI protocol once (1TAI group), twice (2TAI group) or three times (3TAI group) with a 32 days of interval between inseminations. In the 1TAI and 2TAI groups, the artificial insemination was followed by natural service until the end of the breeding period. The conception rate at the first FTAI reached 64.0% (288/450) for the 1TAI, 66.0% (198/300) for the 2TAI and 65.4% (494/755) for the 3TAI groups (P > 0.05). As a result, the final percentage of cows pregnant during the breeding season was lower (P < 0.05) for 1TAI (77.1%; 347/450) than for 2TAI (86.3%; 259/300) and 3TAI (87.4%; 660/755) groups. Overall, the cost per pregnancy ended up being lower for both the 2TAI (R$ 84.53) and 3TAI (R$ 85.20) groups than for the 1TAI group (R$ 95.18). Moreover, the use of three consecutive FTAI with 32 interval between inseminations results in satisfactory efficiency in terms of reproductive performance, without the use of clean-up bulls. Such breeding programs enable producers to conduct a 64-day breeding season with most offspring genetics coming from superior AI sires ( Crepaldi *et al*., 2014 ).

Another recent study evaluated the economic return of cow-calf operation systems (Sá Filho, 2018; Alta Genetics, Uberaba, MG, Brazil; personal communication). In this evaluation, Sá Filho took into account both direct and indirect costs. The monthly cost of the mature cow (R$/head/month) was resulting from the general cost of producing a calf divided by the number of calves weaned and finally divided by a production cycle of 12 months. This value was multiplied by the intercalving interval of a given herd considering only the productive females. Then, the final average cost per calf in the methodology considered the inclusion of monthly costs, including most relevant input and output variables such as deaths and sales of discarded animals or surplus production. Thus, this study evaluated the economic return for cow-calf operation systems under 4 reproductive management scenarios: 1) natural service (1 bull for 30 cows); 2) 1 FTAI (52% conception rate) followed by natural service (1 bull for 35 cows); 3) 2 FTAI (52 and 47% conception rate, respectively) followed by natural service (1 bull for 60 cows); 4) 3 FTAI (52, 45 and 40% conception rate, respectively).

**Figure 2:**
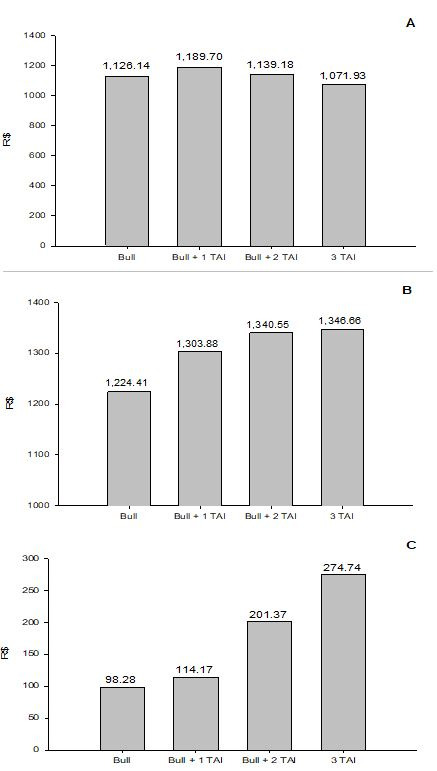
Analysis of the cost per calf weaned (A; R$/calf), revenues per calf (B; R$/calf) and gross profit per calf (C; R$/calf) according to the reproductive management (Natural service, 1-FTAI followed by natural service, 2-FTAI followed by natural service and 3-FTAI). From Sá Filho, 2018; Alta Genetics, Uberaba, MG, Brazil; personal communication.

As shown in Fig. 2 , the highest economic return per calf produced was achieved with 3 FTAI reproductive program. In contrast, the smallest economic return was verified with the natural service. These findings clear indicate that implementing a FTAI program followed by resynchronization is doable and could represent an interesting alternative to produce a greater proportion of calves with a superior genetic value (calves bred through artificial insemination), which certainly will yield more revenue for cow-calf beef operations.

## Use of FTAI in dairy herds

As extensively described in the literature, reproductive performance has a tremendous impact on dairy profitability. Thus, producers and consultants obviously tend to drive their decision-making process based on economics aspects. Although it is fairly well accepted that better reproductive performance is associated with better margins and profits, it is rather difficult to clearly pinpoint the actual value of better fertility in dairy herds. More importantly, producers are somewhat resistant to invest in better reproductive management because the return of investment are not readily seen, and it generally takes about 1 to 2 full years so producers can actually notice a real impact on their milk check and culling strategies. To make it more complex, herds not utilizing data record keeping systems can hardly conclude whether improving reproduction actually improves their profits, and those herds tend to be even more skeptical towards investing in reproductive technologies. Unfortunately, most dairy herds in Brazil have poor record-keeping systems, which makes evidence-based decisions pretty challenging to dairy consultants.

Despite of that, it is clear that high milk production is associated to high rates of steroid metabolism and liver hormonal clearance, decreasing estrus detection efficiency ( Lopez *et al*., 2004 ). In fact, Lopez *et al*. (2004) described in an elegant study that higher producing cows have much shorter receptivity periods during estrus events, which in many cases will last less than 2 h – making visual estrus detection barely impossible. In addition to that, about 30% of lactating cows are not cycling by 60 days in milk due to negative energy balance and other physiological constraints in the postpartum period. Dairy herds in Brazil generally have a greater proportion of *Bos indicus* breed (Gir x Holstein crossbreeding), and postpartum anestrus is even more common and normally affecting 50% of more cows by the end of the voluntary waiting period. Altogether, nowadays the use of FTAI is almost mandatory in modern dairy herds.

**Figure 3:**
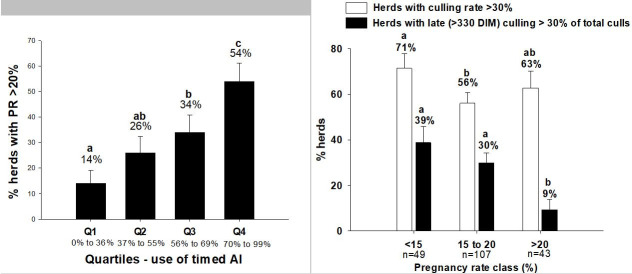
Proportion of dairy herds (n = 200) with acceptable reproductive performance (Pregnancy rate greater than 20%) according to proportion of breedings performed following a FTAI protocol (on the left). Quartiles indicate proportion of FTAI used during the breeding routine. Proportion of herds with culling rates greater than 30% (white bars) and proportion of involuntary cullings (black bars) according to pregnancy rate class.

Based on these facts, in a study performed in 2012 by our research group ( Souza *et al*., 2013 ) in association with the University of Wisconsin (Dr. Wiltbank’s and Dr. Randy Shaver’s labs), we tried to evaluate the impact of FTAI on reproductive efficiency in 200 commercial Holstein herds in Wisconsin. We observed that herds with greater milk production utilized FTAI more aggressively during their reproductive routine compared to lower producing herds, presumably due to lower estrus detection efficiency otherwise. More importantly, herds that used FTAI in a greater proportion of their breedings were more likely to have acceptable reproductive performance (pregnancy rate results above 20%). In addition, greater reproductive performance was not associated with total culling rate, but were not forced to cull cows later in lactation due to non-pregnancy status – as shown in Fig. 3 . Confirming our initial hypothesis that the use of FTAI can overcome some of the physiological limitations for fertility caused by greater milk production and housing type in modern dairy herds.

As shown in Fig. 3 , FTAI will improve pregnancy results in dairy herds, and ultimately will provide a more even chance to higher producing cows to become pregnant during lactation, which would not be the case when visual estrus detection is used. Actually, in the study by Souza *et al*. (2013) , herds in the 1st Quartile, those using less FTAI in their repro routine, had pregnancy rates averaging of 15.8% as compared to herds in the 4th quartile, those using more FTAI, which had pregnancy rates of 19.9%. Thus, the more aggressive use of timed AI represented an increase of roughly 4% points in pregnancy rates or 4 more pregnancies per 100 cows at each reproductive cycle of 21 days. Utilizing the model created by the extension team at the University of Wisconsin, a 4% point increase in pregnancy rate results represents approximately an extra income for commercial herds of US$80 dollars per cow per year (or R$ 280 Brazilian currency).

For Brazilian herds, FTAI adds about R$900 million (~U$278 million) a year mainly by its direct impact on lowering calving intervals and enhancing genetic gain. Most scientific literature shows a reduction in about 20 to 30 days in calving interval, increasing annual milk output in about 10%, accounting for extra 690 million liters of milk out of Brazilian herds or R$759 million (~U$234 million) extra income in Brazilian herd/year. Furthermore, the use of AI sires with superior genetics adds around 300 extra liters of milk per lactation in the future lactating offspring, resulting in an extra income of R$113.9 million (~U$35 million; Baruselli, 2016 ).

## Use of MOET and IVP in dairy herds

Besides FTAI, embryo transfer (ET) can also be strategically utilized to drive higher rates of genetic gain. Even earlier research has described the potential of ET towards greater genetic gain. For example, Nicholas and Smith (1983) reported that an ET program with 1,024 transfers per year in 512 females can boost the rate of genetic improvement some 30% above of that attained with conventional AI using sires selected through the official progeny-testing programme.

**Figure 4:**
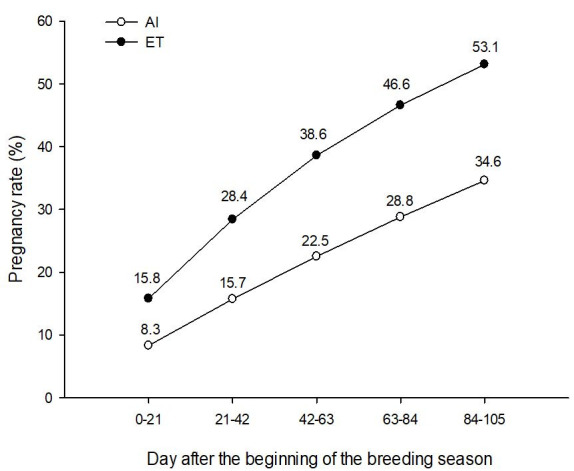
Survival curve assuming 60% service rate, 17% conception rate and 10% pregnancy rate every 21 days in repeat breeders and heat stressed dairy cows during 105 days IA program (pregnancy loss of 19% between 30 and 60 days gestation). For ET program, it was assumed 50% service rate (ET only in recipients with CL), 40% conception rate and 15,3% pregnancy rate every 21 days in repeat breeders and heat stressed dairy cows during 105 days ET program (pregnancy loss of 21% between 30 and 60 days gestation).

Besides to faster genetic progress, ET also has the potential to increase fertility in dairy cows experiencing heat stress ( Putney *et al*., 1989 ; Ambrose *et al*., 1999 ; Baruselli *et al*., 2010 ; Rodrigues *et al*., 2004 , 2007 , 2011 ) and those diagnosed as repeat-breeders ( Dochi *et al*., 2008 ; Rodrigues *et al*., 2011 ), because it bypasses problems associated with fertilization failure and disruption of the oocyte quality in dairy cows ( Ferreira *et al*., 2011 , 2016 ). For example, Baruselli *et al*. 2011, utilizing data from a large commercial herd in Brazil, showed that conception results can be increased in about ~8% (cooler months) to 20% (warmer months) percentage points with the use of ET when compared to AI ( Fig. 4 ). Assuming no change in estrus detection rates at 60%, that represents at least some 7% increase in pregnancy rate results, or according to the Wisconsin model from the ReproMoney program, an extra $US140 dollars per cow per year, and yet not accounting for offspring with better genetics. The scenario for repeat-breeder cows in a comparison between AI and ET is even more dramatic, where conception results found by Baruselli *et al*. 2011 increased in about 15 to 20% points, or using the same rationale, an extra 11% points in pregnancy results or $US220 dollars per cow per year.

A simple simulation model represented in Fig. 4 can illustrate the likely advantage for ET in terms of reproductive efficiency in relation to AI. Thus, data shown in Fig. 4 compares the reproductive efficiency of an AI or ET program in repeat breeders and heat stressed dairy cows utilizing records from earlier scientific publication from our research group that utilized dairy cows in commercial herds. Conception rate results after AI and ET in those studies were approximately 17 and 40%, respectively ( Rodrigues *et al*., 2004 , 2007 ). Then, pregnancy rate following 105 days of breeding period was 34.6% for the AI program and 53.1% for cows under the ET program (53.6% increase in pregnancy rate results). Therefore, we observed that cows subjected to AI had actually greater mean days to conception (59.3 days) than cows exposed exclusively to ET (52.5 days) after the beginning of the reproductive program (7 days saved in terms of days open). Interestingly, Ribeiro *et al*., 2012 compared costs per pregnancy for several breeding programs in US herds including FTAI and ET. Not surprisingly, data from Ribeiro *et al*. (2012) shows that the possibility to produce embryos at reasonably lower costs will have a great impact on the viability to utilize ET in the breeding routine. Thus, choosing the right tool to manage reproduction and leverage fertility with the use of ET is a herd specific decision, that can be certainly implemented considering management and local opportunities that only an experienced veterinarian will be able to interpret with more accuracy.

In later years, the IVP (*in vitro* embryo production) technology became available at commercial level to producers and is rapidly replacing the standard superovulation and flushing platform (MOET) to produce embryos. The advantage of this later technology is the possibility to produce female-sexed embryos without losses related to failure in fertilization and poorer quality commonly reported while utilizing sexed semen in superovulated cows ( Soares *et al*., 2011 ). Hence, IVP is gaining ground compared to standard *in vivo* embryo production mainly because of its greater efficiency in terms of embryo production numbers that can be retrieved out of the same donor cow. Overall, the *in vivo* technology (MOET) produces 5 embryos per procedure per donor at every 45 days. In contrast, the *in vitro* (IVP) can produce 3 embryos per procedure per donor at every 15 days. After one year of embryo production, MOET produces 40 embryos while PIVE produces 72 embryos.

## Conclusion

Currently, commercial herds have plenty of breeding technologies such as FTAI that can be systematically included in the breeding routine to improve reproductive efficiency compared to natural service. The use of resynchronization after first postpartum FTAI, although commonly used in dairy herds, has recently been also proven financially advantageous in beef herds compared to the traditional FTAI followed by natural service by clean-up bulls. These technologies can help change the current scenario of cow-calf production operations in Brazil, which still uses mostly natural service in their breeding programs. Dairy herds also utilize FTAI to overcome low estrus detection efficiency, with clear economic returns since each point in pregnancy rate is estimated to be worth about 15 to 30 US dollars per cow per year. Furthermore, embryo transfer (ET) is an important reproductive technology that can disseminate superior genetics, and potentially improve herd performance. This is a reality particularly to dairy herds, in which ET also has the potential to increase fertility in cows experiencing heat stress and/or in late breeder cows.
